# Ameliorating social anxiety in rural left-behind children through line dance combined with multisensory stimulation

**DOI:** 10.3389/fpsyg.2026.1734053

**Published:** 2026-05-07

**Authors:** Yiping Luo, Xiao Liu, Qian Yang, Jun Tao, Yuqin Sun, Weixin Dong, Jun Chen, Chunxia Lu

**Affiliations:** Department of Sport Education, Hunan Normal University, Changsha city, China

**Keywords:** line dance, multisensory stimulation, randomized controlled trial, rural left-behind children, social anxiety

## Abstract

**Objectives:**

Rural left-behind children in China are at increased risk of social anxiety, which may negatively affect their emotional well-being and social adaptation. This study aimed to examine whether line dance combined with multisensory stimulation could reduce social anxiety in this vulnerable population (R4-C1).

**Methods:**

A randomized controlled trial was conducted with 86 children in grades 5–6 from Huangdu Primary School, Hunan Province. Participants were randomly assigned to four groups: control (CG), line dance only (LDG), line dance with visual stimulation (LVG), and line dance with multisensory stimulation (LMG). The intervention lasted 12 weeks, with sessions held three times per week (Monday, Wednesday, and Friday), followed by a 6-week follow-up period to assess the sustainability of the effects. Social anxiety, fear of negative evaluation, and social avoidance/distress were measured at baseline, post-intervention, and follow-up using validated scales. Data were analyzed using SPSS 27.0. A 4 × 2 repeated-measures ANOVA (group × time) was conducted, with Greenhouse–Geisser correction applied when necessary and Bonferroni-adjusted *post hoc* tests for significant interactions. Statistical significance was set at *p* < 0.05 (R4-C1).

**Results:**

Baseline assessments showed no significant differences among the groups (*p* > 0.05). Significant Group × Time interactions were observed for social anxiety, fear of negative evaluation, and social avoidance/distress (all *p* < 0.01), indicating differential effects of the interventions over time. All intervention groups showed significant reductions in these measures after the 12-week intervention, which were largely maintained at follow-up. The combined line dance and multisensory group (LMG) exhibited the largest and most sustained improvements compared with LDG and LVG (R4-C1).

**Conclusion:**

Line dance, particularly when combined with multisensory stimulation, may represent a promising non-pharmacological approach for reducing social anxiety among rural left-behind children. The findings highlight the potential value of integrating rhythmic movement with sensory enrichment within school-based mental health interventions. Importantly, this study extends the existing evidence on accessible and low-cost psychosocial strategies in resource-limited settings, while also providing practical guidance for educators and policymakers seeking scalable and developmentally appropriate approaches to enhance emotional well-being and social functioning among vulnerable child populations (R4-C1).

## Introduction

In the context of China’s rapid urbanization and large-scale rural-to-urban migration, a substantial number of children have been left behind in rural areas while one or both parents migrate to cities for work. Rural left-behind children (RLBCs) generally refer to minors under 16 years of age who remain in rural households while one or both parents are absent for work-related migration over an extended period (State Council of the People’s Republic of China, 2016) (R5-C1). Parental migration affects a large population of children in rural China, with approximately 6.97 million rural left-behind children reported in recent official statistics, underscoring their importance for child development and public health research (Ministry of Civil Affairs of the People’s Republic of China, 2025). The prolonged absence of parents often disrupts the formation of secure attachment relationships, which are essential for developing confidence in social interactions (Brumariu and Kerns, 2008). With caregiving responsibilities frequently assumed by extended family members, RLBCs often lack consistent emotional support, making them more vulnerable to feelings of rejection, uncertainty, and social withdrawal (Zhao et al., 2017). Moreover, disparities in access to educational and extracurricular opportunities between rural’and urban settings further hinder RLBCs’ social competence and self-efficacy (Su et al., 2013). Such disadvantages may foster feelings of inferiority and insecurity, particularly during interactions with urban peers. Taken together, these factors—rooted in the rural–urban divide—contribute to elevated levels of social anxiety among RLBCs (Wang et al., 2015). Consistent with this, epidemiological studies indicate that the prevalence of social anxiety symptoms in this population ranges from approximately 20 to 40%, exceeding that of their non-left-behind counterparts (Fellmeth et al., 2018; Wang et al., 2019), thereby highlighting their heightened psychosocial vulnerability. This elevated risk underscores the need for targeted, school-based interventions to reduce social anxiety and promote adaptive social functioning (R4-C2) (R5-C1).

Social anxiety is a negative affective state characterized by fear of evaluation, avoidance of social interactions, and physiological symptoms such as blushing, trembling, or rapid heartbeat (Demir et al., 2023). If left unaddressed, persistent social anxiety can impair children’s interpersonal functioning, academic engagement, and long-term psychological adjustment, potentially resulting in depression, loneliness, or suicidal ideation M. Therefore, identifying effective interventions to reduce social anxiety is critical for safeguarding the emotional and developmental well-being of this vulnerable population (R4-C2) (R5-C1).

Empirical evidence suggests that physical activity can alleviate a range of psychological difficulties—including social anxiety, depression, and stress—by improving mood, physiological regulation, and self-efficacy (Goodwin, 2003; Hansen et al., 2001). Among diverse physical activities, line dance has gained increasing recognition as an expressive and rhythmic group-based exercise that integrates body movement, coordination, and shared enjoyment. Compared with traditional forms of exercise, line dance not only promotes physiological activation but also enhances emotional expression and social bonding (Koch et al., 2019). Its inherently social nature—requiring cooperation, synchronization, and collective rhythm—facilitates peer interaction and mutual support, making it particularly suitable for individuals experiencing social difficulties. For RLBCs, participation in line dance may strengthen social connectedness, improve interpersonal communication, and reduce social anxiety through physical engagement and emotional expression (R4-C2).

In parallel, multisensory stimulation—particularly visual and olfactory modalities—has been shown to promote relaxation, positive affect, and emotional regulation (Lindquist and Lange, 2014; Sona et al., 2019; Zhao et al., 2018). This approach may be particularly relevant for children, as multisensory processing plays an important role in perception, cognition, learning, and adaptive behavior across development (Dionne-Dostie et al., 2015). In school settings, multisensory activities may also be feasible to implement because educational environments are inherently multisensory, and sensory-based or sensorimotor approaches are often easier to integrate into school routines than more intensive individualized sensory integration therapies (Broadbent et al., 2018). Evidence indicates that simultaneous visual and olfactory stimulation can reduce psychological stress and enhance well-being by promoting physiological calmness and emotional balance (Herz, 2009). In addition, child-focused research suggests that multisensory input may support attention, learning, and adaptive responding, thereby providing a practical basis for its application in school-aged children (Jeong, 2024; Pfeiffer et al., 2011) (R4-C2) (R5-C1).

Although both line dance and multisensory stimulation have shown potential benefits for emotional and social functioning, existing studies have rarely examined their combined application in rural left-behind children with social anxiety. The integration of line dance and multisensory stimulation is theoretically grounded in the complementary mechanisms of embodied movement and sensory processing. Specifically, line dance may promote social connectedness and active participation through rhythmic and synchronized group activity, whereas multisensory stimulation may facilitate relaxation and emotional regulation. Their combination may therefore provide a more comprehensive intervention framework that addresses both behavioral engagement and affective adjustment (R4-C2) (R5-C1).

Given that line dance emphasizes rhythmic coordination and social engagement, and multisensory stimulation enhances relaxation and affective regulation, integrating these two approaches may provide a comprehensive and synergistic intervention framework. More importantly, this integrated approach may be developmentally appropriate for children and practically applicable in school contexts. For RLBCs, this combined approach could foster both physiological activation and emotional equilibrium, thereby reducing social anxiety and improving psychological adaptation. Accordingly, the present study aimed to examine the effects of line dance combined with multisensory stimulation on social anxiety among rural left-behind children (R4-C2) (R5-C1).

## Methods

### Research design

This study employed a randomized controlled trial (RCT) design to examine the effects of three intervention conditions—Line Dance Only Group (LDG), Line Dance with Unisensory Visual Stimulation Group (LVG), and Line Dance with Multisensory Stimulation Group (LMG)—on social anxiety among rural left-behind children (RLBCs). A no-intervention control group (CG) continued routine school activities throughout the study period. The intervention lasted 12 weeks and was followed by a 6-week follow-up period, resulting in a total study duration of 18 weeks (R3-C1)(R4-C3).

After baseline assessment and eligibility confirmation, participants were randomly assigned to one of the four groups using a computer-generated random sequence, with an equal allocation ratio of 1:1:1:1.(R3-C1)(R4-C3) The allocation sequence was prepared by a researcher who was not involved in participant recruitment, intervention delivery, or outcome assessment. Group assignments were concealed in sealed, opaque, sequentially numbered envelopes and opened only after completion of baseline assessment. Outcome evaluators remained blinded to group allocation throughout the study (R3-C1)(R4-C3). To maintain blinding, several measures were implemented: group assignment information was managed exclusively by a designated research coordinator and inaccessible to evaluators, participants were explicitly instructed prior to each assessment session not to disclose intervention-related information to evaluators, and data collection sessions used standardized procedures and scripts to ensure consistency and reduce the risk of unintentional unblinding (R3-C1).

Written informed consent was obtained from all participants and their legal guardians after detailed explanations of the study objectives, procedures, potential risks, benefits, and alternative options. Participation was voluntary, and all participants were free to withdraw at any time without penalty. Data confidentiality and anonymity were strictly maintained. The study protocol was reviewed and approved by the Ethics Committee of Hunan Normal University (Approval No. 2022 Lun Shen No. 309) in accordance with the Declaration of Helsinki and the Belmont Report. This trial was prospectively registered at the Chinese Clinical Trial Registry (ChiCTR2500098927; registration date: March 17, 2025) (R4-C3).

### Sample size and participants

**Figure 1:**
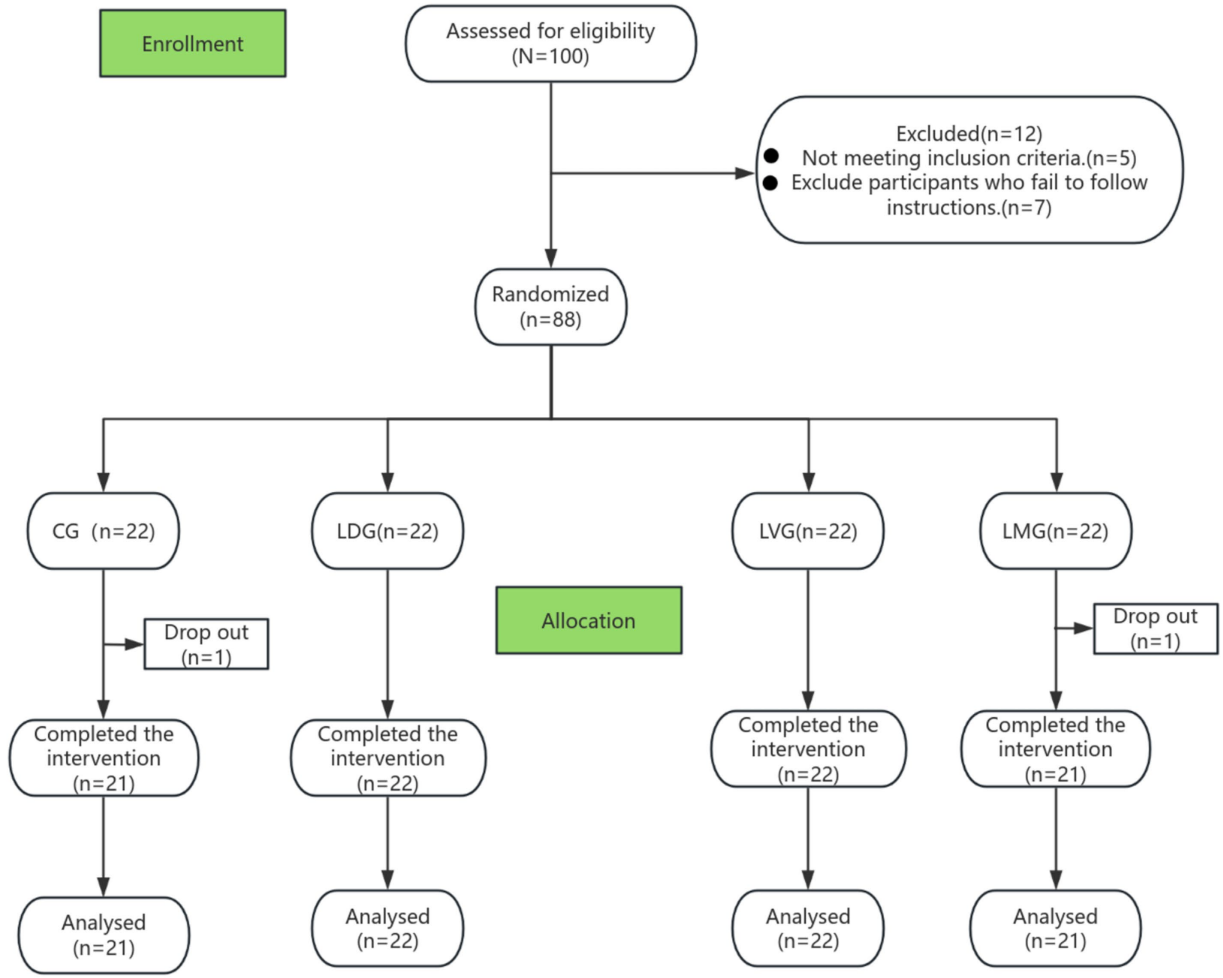
CONSORT diagram.

The required sample size was estimated using G*Power 3.1 software (Faul et al., 2009). According to Cohen’s (1988) classification, an effect size of *f* = 0.30 (medium-to-large), a significance level of *α* = 0.05, and a statistical power of 0.80 were adopted, which are appropriate for behavioral intervention studies with measurable psychological outcomes. Power analysis indicated that a minimum of 72 participants was required. Considering an anticipated attrition rate of approximately 20%, the final target enrollment was increased to 88 participants (22 per group). During the study, two participants withdrew due to illness or scheduling conflicts, resulting in a final sample of 86 participants. Participant flow and attrition are illustrated in the CONSORT diagram (Figure 1).

A total of 86 rural left-behind children aged 10–12 years were recruited from several primary schools in Hunan Province. Following a preliminary screening, Huangdu Primary School in Shaodong City was selected as the study site. Inclusion criteria were as follows: (i) enrollment in Grade 5 or 6 (aged 10–12 years); (ii) classification as rural left-behind children (defined as residing in rural households while one or both parents have worked away from home for at least six consecutive months); (iii) a score ≥8 on the Social Anxiety Scale for Children (La Greca and Stone, 2010); and (iv) voluntary participation with written informed consent provided by the child and their legal guardian. Exclusion criteria included: (i) physical or neurological disorders, or any medical contraindication to exercise; (ii) current participation in psychological counseling or treatment involving psychotropic medications; (iii) involvement in other structured physical training or extracurricular sports programs during the study period; (R5-C4) (iv) Children with known fragrance or olfactory sensitivities or allergies; (R5-C3) or (v) inability or unwillingness to adhere to the 18-week protocol.

## Measures

### Social demographic questionnaire

The socio-demographic questionnaire was completed jointly by the children and their guardians to obtain information on the participants’ family and living conditions. The questionnaire collected data on the child’s gender, age, only-child status, parental marital status, frequency of parental migration, frequency of parents returning home, parental occupation, and annual household income. These variables were used to describe sample characteristics and assess baseline comparability across groups (R4-C3).

### Social anxiety scale for children (SASC)

The Social Anxiety Scale for Children (SASC), developed by La Greca and colleagues, was used to assess social anxiety levels among rural left-behind children (RLBCs) (La Greca and Lopez, 1998).(R4-C3) The SASC is designed for children and adolescents aged 7–16 years and consists of 10 items categorized into two subscales: *Fear of Negative Evaluation* (FNE) and *Social Avoidance and Distress* (SAD). Each item is rated on a 3-point Likert scale (“0” = never, “1” = sometimes, “2” = often), yielding a total score ranging from 0 to 20. According to the original scoring criteria, a total score of ≥8 indicates the presence of social anxiety, with higher scores reflecting greater symptom severity. The Chinese version of the SASC, which has been translated and psychometrically validated in large samples of Chinese children, demonstrates satisfactory reliability and validity (Mo and Bai, 2022). In the present study, this version was used to evaluate social anxiety among Chinese RLBCs, showing good internal consistency (Cronbach’s *α* = 0.721) and sampling adequacy (KMO = 0.813).

### Intervention

All intervention groups participated in a standardized line dance program over a 12-week period. Sessions were conducted three times per week—on Monday, Wednesday, and Friday from 16:10 to 17:10—and each group met at the same scheduled time of day to ensure consistency. Each session consisted of four phases: (1) 10-min warm-up with light aerobic exercises, (2) 20-min skill acquisition phase through age-appropriate dance steps to develop rhythmic coordination, balance, and spatial awareness, (3) 25-min choreographed routine practice of continuous choreographed dances performed in group synchronization with steady background music (100–120 BPM), (4) and 5-min cool-down involving stretching and relaxation. Exercise intensity was maintained at a moderate level (40–59% heart rate reserve, HRR) (Garber et al., 2011) (R5-C6) and monitored using Polar heart rate monitors, with additional manual spot checks of radial pulse (R4-C3)(R5-C4).

**Figure 2:**
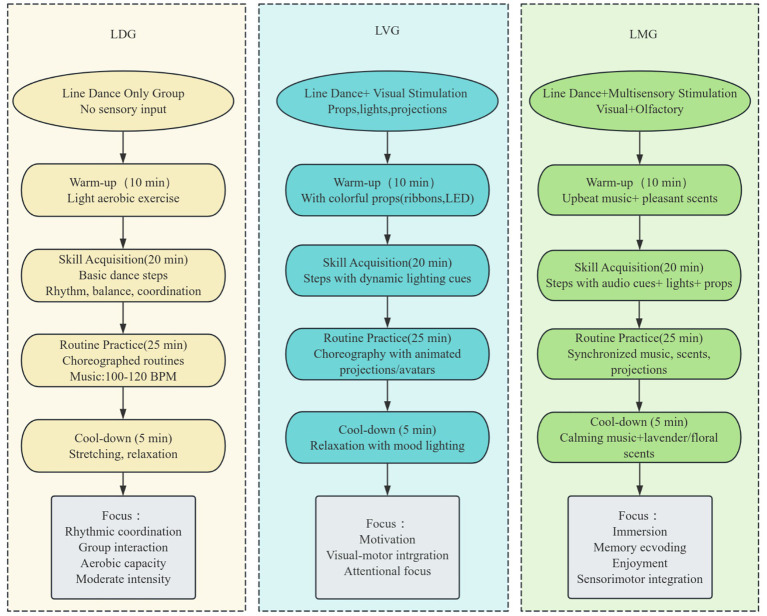
Flowchart of intervention protocols across study groups.

To maintain intervention fidelity, session frequency, timing, duration, dance content, instructional progression, and exercise intensity were standardized across groups (see Table 1 and Figure 2). All sessions were delivered by trained instructors following the same intervention manual and were supervised by the research team throughout the study. Children in the intervention groups continued their routine school activities; however, participation in other structured physical training or extracurricular sports programs during the study period was restricted and monitored in accordance with the study exclusion criteria (R3-C4)(R4-C3)(R5-C5) (R5-C7). The four groups differed only in the type of sensory stimulation provided during the intervention (R3-C4)

**Table 1:** Overview of intervention conditions and sensory stimuli.

Group	Sensory modalities	Warm-up (10 min)	Skill acquisition (20 min)	Dance routine practice (25 min)	Cool-down (5 min)
CG	None (regular PE)	Usual PE activities	Varies	Varies	Varies
LDG	None	Light aerobic activities (marching, arm circles, dynamic stretching)	Basic line dance steps (grapevine, step-touch, box steps, knee lifts)	Choreographed routines, steady music (100–120 BPM)	Walking, static stretching, breathing
LVG	Visual (props, lights, projections)	Warm-up with colorful props (ribbons, LED bracelets)	Steps practiced with dynamic lighting synced to music	Routines with animated projections/avatars as movement cues	Relaxation with visual mood lighting
LMG	Visual + Olfactory	Warm-up with upbeat music (110–130 BPM) and pleasant scents	Steps with rhythmic audio cues (claps, prompts), props, and lights	Routines with synchronized music, scents, and visual projections	Stretching with calming music and lavender/floral scents

#### Control group (CG)

Children in the control group continued their regular school physical education (PE) classes during the 18-week intervention period. PE sessions typically included a variety of age-appropriate activities such as calisthenics, ball games, running, and free play, but no structured line dance or additional sensory stimulation was provided. Exercise intensity and content varied according to the school curriculum and were not systematically monitored.

#### Line dance only group (LDG)

Children in the LDG engaged in the standardized line dance program without additional sensory stimulation. The intervention focused on rhythmic coordination, group interaction, and aerobic participation through synchronized movement performed with steady background music (100–120 BPM).(R4-C3)

#### Line dance with unisensory visual stimulation group (LVG)

Children in the LVG performed the same standardized line dance program as the LDG, but with additional visual stimulation to enhance attention and engagement. Visual inputs included colorful props (e.g., ribbons, scarves, or LED bracelets) (Shams and Seitz, 2008; Stein and Stanford, 2008), dynamic lighting synchronized with music, and animated wall projections or avatars providing movement cues during routine practice. The cool-down phase also incorporated visual mood lighting (R4-C3).

#### Line dance with multisensory stimulation group (LMG)(R4-C3)

Children in the LMG followed the same standardized line dance program as the LDG, with additional multisensory stimulation. Alongside visual inputs such as props, lighting, and projections, olfactory stimuli were delivered using a standardized diffusion procedure with mild, tolerable intensity. Pleasant scents were used during the warm-up and practice phases, and calming music with subtle lavender or floral scents was incorporated during the cool-down phase (Rachel S Herz, 2009; Zatorre et al., 2007).

To ensure safety and feasibility, children were screened for fragrance-related allergies or sensitivities based on guardian reports and school health records prior to participation. All sensory materials were age-appropriate, non-toxic, and applied at safe levels. A pilot test was conducted to assess the acceptability of the sensory stimuli, during which no adverse reactions were observed. Throughout the intervention, participants were continuously monitored by trained instructors, and no sensory-related adverse events were reported (R4-C3)(R5-C3).

### Statistical analyzes

All statistical analyses were conducted using SPSS version 27.0. The normality of continuous variables was assessed using the Shapiro–Wilk test. Baseline differences among the four groups were examined using one-way analysis of variance (ANOVA) for normally distributed continuous variables, the Kruskal–Wallis test for non-normally distributed continuous variables, and the chi-square test for categorical variables. For variables that did not meet the normality assumption, non-parametric methods were applied. Data transformation was not performed in order to preserve the original scale and interpretability of the measures (R4-C4).

For the main intervention analyses, normally distributed outcomes were analyzed using a 4 × 2 repeated-measures ANOVA, with group (CG, LDG, LVG, and LMG) as the between-subjects factor and time (pre-test and post-test) as the within-subjects factor. Main effects of group and time, as well as the Group × Time interaction, were examined. When Mauchly’s test indicated a violation of the sphericity assumption, the Greenhouse–Geisser correction was applied. If the Group × Time interaction was significant, Bonferroni-adjusted *post hoc* comparisons were performed. If the interaction was not significant, the main effects were interpreted. For repeated-measures ANOVA, partial eta squared (*ηp*^2^) was reported as the effect size index in accordance with standard practice (R4-C3)(R4-C4).

For outcomes that did not meet the assumption of normality, generalized estimating equations (GEE) were used to examine the effects of group, time, and their interaction across the four groups. This method was chosen because it is robust to violations of normality and suitable for repeated-measures data. All tests were two-tailed, and statistical significance was set at *p* < 0.05.(R4-C4).

## Results

### Baseline characteristics of participants

A total of 86 participants had a mean age of 10.39 ± 0.87 years and reported generally high levels of social anxiety (11.66 ± 2.69). Males accounted for 56% of the sample. Regarding family background, 22% of participants had divorced parents, 24% had parents working away from home, and 23% had parents who returned home only once per year. At baseline, the CG, LDG, LVG, and LMG showed no significant differences in sociodemographic characteristics or social anxiety levels (all *p* > 0.05). Detailed participant characteristics are presented in Table 2 (R4-C5) (R3-C2.3).

**Table 2:** Comparison of general information among the four groups (M ± SD, *n* (%)).

Variable	Characteristic	CG (*n* = 21)	LDG (*n* = 22)	LVG (*n* = 22)	LMG (*n* = 21)	*F/*χ^2^	*p*
Gender	Male	12 (57.1)	12 (54.5)	12 (54.5)	12 (57.1)	0.059	0.99
	Female	9 (42.9)	10 (45.5)	10 (45.5)	9 (42.9)		
Age		10.57 ± 0.75	10. 18 ± 0.91	10. 18 ± 0.85	10.62 ± 0.97	1.613	0.193
Whether it is an only child	True	14 (66.7)	13 (59.1)	16 (72.7)	14 (66.7)	0.921	0.82
	No	7 (33.3)	9 (40.9)	6 (27.3)	7 (33.3)		
Parental marital status	Married	14 (66.7)	13 (59.1)	14 (63.6)	12 (57.1)	1.262	0.99
	Divorced	4 (19.0)	5 (22.7)	5 (22.7)	5 (23.8)		
	Digamy	2 (9.55)	3 (13.6)	2 (9.1)	2 (9.5)		
	Widowed	1 (4.8)	1 (4.5)	1 (4.5)	2 (9.5)		
Parental outings	Father outing	10 (47.6)	9 (40.9)	14 (63.6)	11 (52.4)	2.994	0.81
	Mother outing	6 (28.6)	7 (31.8)	4 (18.2)	4 (19.0)		
	Parents outing	5 (23.8)	6 (27.3)	4 (18.2)	6 (28.6)		
Parental frequency of returning home	Once a week	2 (9.5)	2 (9.1)	2 (9.1)	2 (9.5)	1.579	0.99
	Once a month	3 (14.3)	4 (18.2)	2 (9.1)	2 (9.5)		
	Once every 6 months	11 (52.4)	11 (50.0)	12 (54.5)	13 (61.9)		
	Once a year	5 (23.8)	5 (22.7)	6 (27.3)	4 (19.0)		
Parents’ careers	Farm	4 (19.0)	4 (18.2)	4 (18.2)	4 (19.0)	1.106	0.99
	Work	12 (57.1)	14 (63.6)	14 (63.6)	13 (61.9)		
	Self-employed	3 (14.3)	3 (13.6)	3 (13.6)	2 (9.5)		
	Others	2 (9.5)	1 (4.5)	1 (4.5)	2 (9.5)		
Household income (Yuan/year)	>10,000	3 (14.3)	2 (9.1)	2 (9.1)	3 (14.3)	1.185	0.99
	>30,000	6 (28.6)	7 (31.8)	7 (31.8)	6 (28.6)		
	>50,000	10 (47.6)	11 (50.0)	12 (54.5)	10 (47.6)		
	>80,000	2 (9.5)	2 (9.1)	1 (4.5)	2 (9.5)		
Social anxiety	SASC	11.43 ± 1.94	11.73 ± 3.00	12.00 ± 2.90	11.48 ± 2.94	0.20	0.90
	FNE	6.52 ± 1.60	6.82 ± 1.99	7.05 ± 2.26	6.29 ± 2.17	0.58	0.63
	SAD	4.90 ± 1.26	4.91 ± 2.02	4.95 ± 1.43	5.19 ± 1.33	0.16	0.92

### Efficacy on social anxiety symptoms

Consistent patterns of intervention effects were observed across all three social anxiety outcomes (SA, FNE, and SAD). At baseline (T_0_), no significant differences were found among the four groups (all *p* > 0.05), indicating comparable starting points for subsequent analyses.

Repeated-measures ANOVA revealed significant Group × Time interactions for all three outcomes (SA: *F* (3, 82) = 34.354, *p* < 0.01, *η_p_*^2^ = 0.557; FNE: F (3, 82) = 26.899, *p* < 0.01, *η_p_*^2^ = 0.496; SAD: F (3, 82) = 13.952, *p* < 0.01, *η_p_*^2^ = 0.338), along with significant main effects of Time (SA: *F* (1, 82) = 197.190, *p* < 0.01, *η_p_*^2^ = 0.706; FNE: F (1, 82) = 178.406, *p* < 0.01, *η_p_*^2^ = 0.685; SAD: F (1, 82) = 58.195, *p* < 0.01, *η_p_*^2^ = 0.415). These findings indicate that changes in social anxiety symptoms over time differed significantly across experimental conditions (R3-C2.1)(R5-C7). From T_0_ to T_1_, the control group (CG) showed no significant changes in any outcome (all *p* > 0.05). In contrast, all three intervention groups (LDG, LVG, and LMG) demonstrated significant reductions in SA, FNE, and SAD (all *p* < 0.01). The magnitude of reduction showed a clear gradient across groups, with LMG exhibiting the largest decreases, followed by LVG and LDG (R4-C6).

Notably, the LMG yielded the greatest and most sustained reductions across all outcomes, achieving the lowest post-intervention scores at T_1_. *Post hoc* comparisons further clarified these differences: both LVG and LMG showed significantly lower scores than CG at T1 (*p* < 0.05), while LMG performed significantly better than both LDG and LVG (*p* < 0.05). This pattern suggests that the combined multisensory intervention produced superior effects compared to single-modality interventions (R4-C6)(R5-C7).

**Figure 3:**
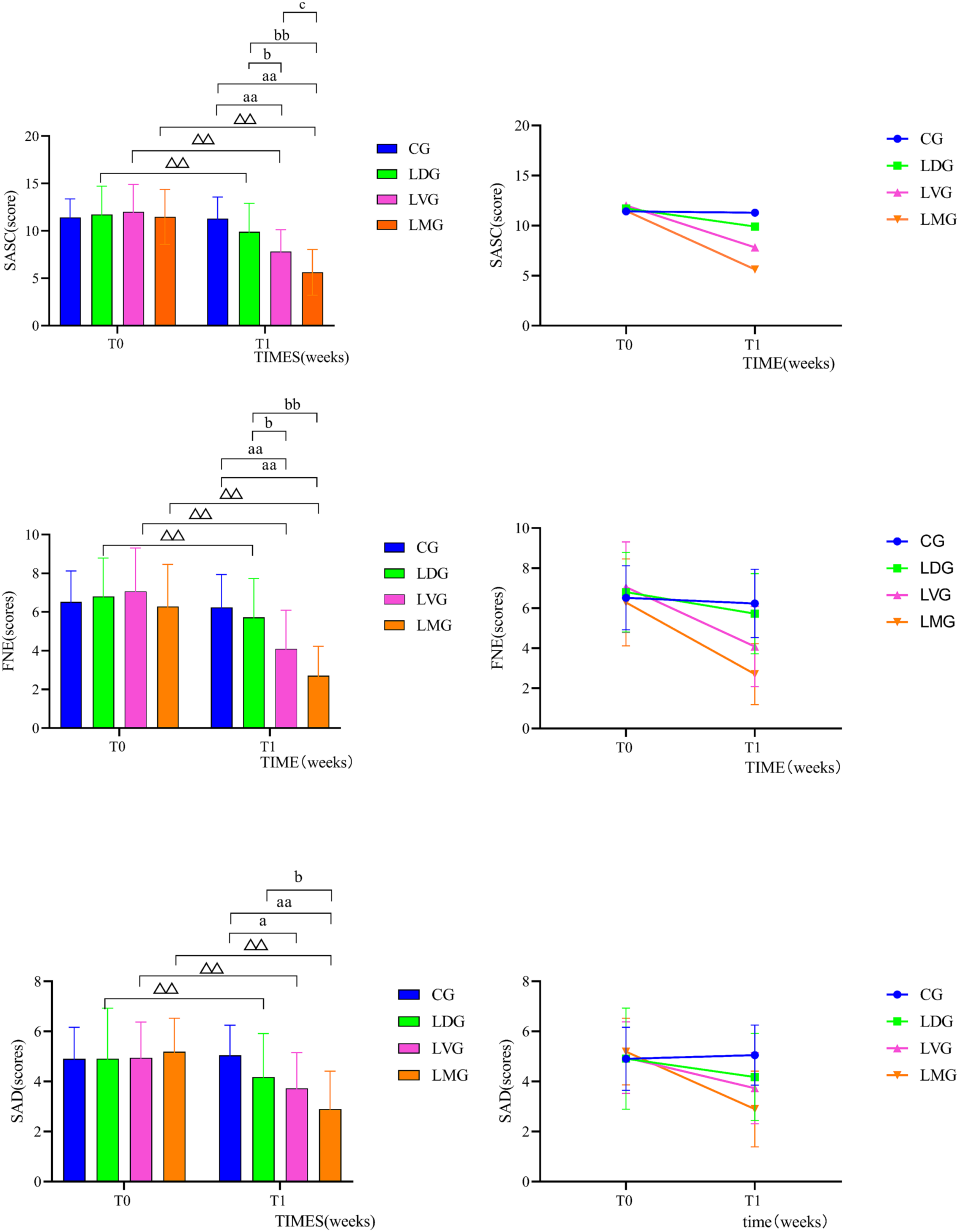
Efficacy on social anxiety symptoms.

Regarding the main effects of group, significant between-group differences were observed for SA (*F*(3, 82) = 5.376, *p* < 0.01, *η_p_*^2^ = 0.164) and FNE (*F*(3, 82) = 4.897, *p* < 0.01, *η_p_*^2^ = 0.152), whereas no significant group effect was found for SAD (*F*(3, 82) = 1.681, *p* > 0.05, *η_p_*^2^ = 0.058). Overall, these findings indicate that group-based movement interventions are effective in reducing multiple dimensions of social anxiety in children, with the multisensory approach demonstrating the greatest benefit. Detailed descriptive statistics are presented in Table 3 and Figure 3 (R4-C5).

**Table 3:** Pre-test and post-test data of dependent variable index in experiment.

variable	Group	T_0_	T_1_	*F*/*η_p_^2^*/*p* group	*F*/*η_p_^2^*/*p* time	*F*/*η_p_^2^*/*p* group*time
SA	CG	11.43 ± 1.94	11.29 ± 2.28	5.376/0. 164/<0.01^**^	197.190/0.706/<0.001^***^	34.354/0.557/<0.001^***^
	LDG	11.73 ± 3.00	9.91 ± 2.99^△△^			
	LVG	12.00 ± 2.90	7.82 ± 2.30^△△aab^			
	LMG	11.48 ± 2.94	5.62 ± 2.42^△△aabbc^			
FNE	CG	6.52 ± 1.60	6.24 ± 1.70	4.897/0. 152/<0.01^**^	178.406/0.685/<0.001^***^	26.899/0.496/<0.001^***^
	LDG	6.82 ± 1.99	5.73 ± 2.00^△△^			
	LVG	7.05 ± 2.26	4.09 ± 2.00^△△aab^			
	LMG	6.29 ± 2.17	2.71 ± 1.52^△△aabb^			
SAD	CG	4.90 ± 1.26	5.05 ± 1.20	1.681/0.058/>0.05	58.195/0.415<0.001^***^	13.952/0.338/<0.001^***^
	LDG	4.91 ± 2.02	4.18 ± 1.74^△△^			
	LVG	4.95 ± 1.43	3.73 ± 1.42^△△a^			
	LMG	5.19 ± 1.33	2.90 ± 1.51^△△aab^			

Compared with T_0_, ^△^<0.05, ^△△^<0.001; compared with CG, *a* < 0.05; compared with LDG, *b* < 0.05; compared with LVG, c < 0.005; **p < 0.01, ***p < 0.001; SASC, social anxiety; FNE, afraid of being denied; SAD, social avoidance.

## Discussion

The present study examined the effects of a line dance–based intervention on social anxiety among rural left-behind children (RLBCs) under three experimental conditions: line dance only (LDG), line dance combined with unisensory visual stimulation (LVG), and line dance integrated with multisensory (visual and olfactory) stimulation (LMG). The results showed that all three interventions significantly reduced overall social anxiety, fear of negative evaluation, and social avoidance and distress compared with the control group, suggesting that embodied and socially engaging physical activity may serve as an effective non-pharmacological approach to improving emotional and social well-being in RLBCs (R4-C7).

The LDG produced significant but relatively modest improvements in social anxiety symptoms. This finding is consistent with previous studies showing that rhythmic dance interventions can reduce anxiety and enhance mood through physical activation and social participation (Bennett et al., 2021; Feenstra et al., 2022). However, compared with the sensory-enhanced conditions, line dance alone may provide more limited support for attentional and emotional regulation, which may explain its comparatively smaller effect (R4-C7).

The LVG showed greater reductions in fear of negative evaluation and social avoidance than the LDG. This may be related to the addition of visual elements, which increased environmental structure, movement coordination, and social predictability (Bigliassi et al., 2018). By directing attention toward external cues and shared group activity, visual stimulation may help reduce self-focused rumination and ease anxiety in socially evaluative situations. Nevertheless, because this condition involved only one sensory modality, its regulatory effect may have remained less comprehensive than that of the multisensory intervention (R4-C7).

Among the three intervention groups, the LMG produced the greatest and most sustained reductions in social anxiety. This finding suggests that integrating rhythmic movement with multisensory stimulation may yield additional benefits beyond those associated with line dance alone or combined with visual stimulation. Multisensory enrichment has been shown to facilitate emotional regulation, enhance attentional engagement, and promote relaxation (Sona et al., 2019; Thepsatitporn et al., 2024), which may collectively contribute to improved psychological outcomes (R3-C3).

These effects may be particularly salient for rural left-behind children (RLBCs), who often experience reduced emotional security and lower confidence in social contexts due to prolonged parental absence (Fan et al., 2010; Fellmeth et al., 2018). In this population, enriched, supportive, and predictable intervention environments may help foster emotional comfort and social connectedness, while also enhancing perceived self-efficacy and adaptive responses during group participation. From a developmental psychology perspective, such benefits are consistent with theoretical frameworks such as attachment theory and self-efficacy theory, which emphasize the role of structured, supportive experiences in promoting emotional security and social competence Locke (R5-C7).

In addition, strict safety procedures were implemented for the sensory components, including pre-screening for allergies, pilot testing of stimuli, and continuous monitoring during sessions. No adverse effects were observed, supporting the safety and feasibility of multisensory stimulation in school-based settings (R5-C7).

## Limitations and future directions

Despite these encouraging findings, several limitations should be acknowledged. First, the sample was drawn from a single province (Hunan), which may limit the generalizability of the results. Future multisite studies with larger and more diverse samples are needed to strengthen external validity. Second, only visual and olfactory stimuli were included, whereas other sensory modalities, such as tactile and auditory cues, may also contribute to emotional regulation. Future research should further examine the effects of different sensory modalities within embodied interventions. Third, individual differences, including baseline anxiety, gender, sensory sensitivity, duration of being left behind, and parental return frequency, were not controlled and may influence children’s social anxiety and responsiveness to intervention. Future research should adopt controlled and longitudinal designs to examine the role of these individual differences, develop targeted intervention strategies, and expand the scope of individual variables to enhance research rigor and intervention effectiveness (R5-C2). Longer follow-up periods are also needed to determine the sustainability of intervention effects over time (R4-C8)(R5-C3).

## Practical implications

From a practical perspective, the findings suggest that line dance combined with multisensory stimulation may offer a cost-effective, culturally adaptable, and scalable approach to supporting the emotional health of rural left-behind children. This integrated approach is consistent with school-based mental health promotion frameworks and may be implemented in rural schools or community settings with relatively limited equipment and professional supervision (R4-C8)(R5-C7).

Introducing rhythmic, group-based activities such as line dance into daily school routines may help strengthen social connectedness and emotional expression among children experiencing prolonged parental separation (Fong Yan et al., 2024; Li et al., 2025). The addition of multisensory elements, such as visual and olfactory cues, may further enhance relaxation and stress regulation in low-resource settings (Thepsatitporn et al., 2024). With appropriate teacher and caregiver training, this approach may also be extended to family-or community-based programs and adapted for broader implementation in underserved regions. Future translational research should further evaluate intervention fidelity, cost-effectiveness, and cultural adaptability to support sustainable application (R4-C8)(R5-C7).

## Conclusion

This study provides empirical evidence that line dance–based interventions can effectively reduce social anxiety among rural left-behind children. All three intervention modes—line dance alone (LDG), line dance with unisensory visual stimulation (LVG), and line dance with multisensory visual and olfactory stimulation (LMG)—significantly alleviated social anxiety symptoms compared with the control group. However, the magnitude of improvement varied across interventions: line dance alone produced the smallest yet meaningful reduction, line dance with visual stimulation achieved greater benefits by enhancing attention focus and social coordination, and the multisensory condition yielded the most substantial and sustained decrease in social anxiety.

## Data Availability

The raw data supporting the conclusions of this article will be made available by the authors, without undue reservation.
